# Editorial: Mesenchymal and immune cell crosstalk in fibrotic diseases

**DOI:** 10.3389/fimmu.2023.1304773

**Published:** 2023-10-11

**Authors:** Kinji Asahina, Subburaj Ilangumaran

**Affiliations:** ^1^ Central Research Laboratory, Shiga University of Medical Science, Otsu, Japan; ^2^ Department of Immunology and Cell Biology, Faculty of Medicine and Health Sciences, Université de Sherbrooke, Sherbrooke, QC, Canada

**Keywords:** asthma, exosomes, fibroblasts, fibrosis, hepatic stellate cells, Kupffer cells, macrophages, skin

Fibroblasts, a type of mesenchymal cells, are a source of myofibroblasts and are key players in organ fibrosis. Upon organ injury, tissue-resident fibroblasts are activated to differentiate into myofibroblasts (1). Myofibroblasts express extracellular matrix proteins and participate in progression of fibrosis in the liver, lungs, skin, and other organs. Myofibroblasts produce pro-inflammatory cytokines and chemokines, and induce recruitment of inflammatory immune cells to the fibrotic area. Although fibrosis is recognized as a wound healing process, excessive accumulation of extracellular matrix proteins and myofibroblasts in tissues results in severe fibrosis, which is considered irreversible. Currently, there are no approved drugs for reversal of severe organ fibrosis.

In liver fibrosis, hepatic stellate cells are the major source of myofibroblasts (2). Hepatic stellate cells store vitamin A lipids and reside in the space of Disse between hepatocytes and sinusoidal endothelial cells. Following liver injury, damaged hepatocytes release damage-associated molecular patterns and induce activation of hepatic stellate cells. The activated hepatic stellate cells synthesize extracellular matrix proteins, pro-inflammatory cytokines, and chemokines to induce inflammation. In this Research Topic, Liu et al. have reviewed recent findings on the role of exosomes in liver fibrosis. Exosomes derived from hepatocytes or macrophages induce activation of hepatic stellate cells in liver fibrosis. In contrast, exosomes derived from mesenchymal stem cells exert antifibrotic effects on the activated hepatic stellate cells. Exosomes may serve as biomarkers for diagnosis of liver diseases, including fibrosis.

Tissue-resident macrophages are derived from myeloid progenitor cells present in the embryonic yolk sac (3). In the liver, resident macrophages, called Kupffer cells, play an important role in protecting the liver from microorganisms that are transported from the intestine through the portal vein. Kupffer cells communicate with hepatic stellate cells and hepatocytes via soluble factors to control liver homeostasis, response to injury, and regeneration. In addition to Kupffer cells, monocyte-derived macrophages are recruited to the injured liver tissue to elicit inflammatory responses (4). Hassan et al. have reviewed the roles of resident Kupffer cells and monocyte-derived macrophages in liver injury. Both the cell types exhibit distinct phenotypes and contribute toward inflammation, fibrogenesis, and repair during different phases. Activation of tissue repair macrophages or suppression of inflammatory macrophages is an attractive therapeutic strategy for treatment of liver fibrosis. By modulating these cells, resolution of fibrosis and regeneration of the liver can be controlled. SOCS1 is a negative regulator of many signaling pathways activated by cytokines and growth factors. Kandhi et al. experimentally deleted SOCS1 in hepatic stellate cells and observed overactivation of these cells and induction of severe liver fibrosis. Deletion of SOCS1 in hepatic stellate cells resulted in recruitment of inflammatory macrophages to the fibrotic liver, indicating the crucial role of hepatic stellate cells in the recruitment of inflammatory macrophages in liver fibrosis.

Similar to hepatic stellate cells in the liver, fibroblasts in other organs play an important role in fibrotic reactions. Asthma is a chronic inflammatory lung disease in which lung fibroblasts secrete inflammatory cytokines to induce inflammation. Neutrophils, eosinophils, mast cells, and T cells are major immune cells involved in asthmatic inflammation. Thiam et al. reviewed co-culture systems of lung fibroblasts and immune cells in 2D and 3D. These new culture systems may help clarify crosstalk between fibroblasts and immune cells and identify new therapeutics for the treatment of asthma and fibrosis in other organs.

In internal organs, tissue fibroblasts originate from mesodermal cells. In the liver, hepatic stellate cells express both mesenchymal and neural cell markers; however, they are derived from the mesoderm during embryonic development (5). In contrast, the origins of dermal fibroblasts in the skin are heterogeneous; they are derived from the mesoderm and neural crest. Knoedler et al. have reviewed the heterogeneous embryonic origins of dermal fibroblasts and their distinct roles in wound healing process in the skin.

Similar to dermal fibroblasts, mesenchymal populations in other organs, such as the liver, are heterogeneous (6). Further studies are required to understand whether heterogeneous mesenchymal populations play distinct roles in organ fibrosis and inflammation. As evidenced in this Research Topic, crosstalk between fibroblasts and immune cells, such as macrophages, is an attractive target for suppression of fibrosis. However, macrophages are heterogeneous populations that rapidly change their phenotype depending on their environment and stimuli received from other cells. Further comprehensive studies on the crosstalk between mesenchymal cells and macrophages is necessary to develop therapeutics against organ fibrosis.

## Author contributions

KA: Writing – original draft. SI: Writing – original draft.
